# Creating a ‘choose your topic’ massive open online course: an innovative and flexible approach to delivering injury prevention education

**DOI:** 10.1080/10872981.2021.1955646

**Published:** 2021-07-20

**Authors:** Nadine I. Ibrahim, Lauren Bohm, Jessica S. Roche, Sarah A. Stoddard, Rebecca M. Quintana, Jennifer Vetter, Jeffrey Bennett, Beth Costello, Patrick M. Carter, Rebecca Cunningham, Andrew N. Hashikawa

**Affiliations:** a Department of Otolaryngology, Head and Neck Surgery, Michigan Medicine, Ann Arbor, MI, USA; b University of Michigan Injury Prevention Center, University of Michigan, Ann Arbor, MI, USA; c Department of Emergency Medicine, Michigan Medicine, Ann Arbor, MI, USA; d Department of Systems, Populations and Leadership, School of Nursing, University of Michigan, Ann Arbor, MI, USA; e Center for Academic Innovation, University of Michigan, Ann Arbor, MI, USA; f Department of Health Behavior & Health Education, School of Public Health, University of Michigan, Ann Arbor, MI, USA; g University of Michigan Office of Research, Ann Arbor, MI, USA; h Department of Pediatrics, Michigan Medicine, Ann Arbor, MI, USA

**Keywords:** Pediatric injury prevention, medical education, anticipatory guidance, Massive Open Online Course (MOOC), Small Private Online Course (SPOC)

## Abstract

**Background:**

A pediatric injury prevention course has not been available as a massive open online course (MOOC). Creating a comprehensive topic course is particularly challenging because the traditional, week-by-week linear curriculum design is often a barrier to learners interested in only specific topics. We created a novel, flexible course as both a ‘choose your topic’ MOOC for the public learner and a Small Private Online Course (SPOC) for medical students.

**Methods:**

We describe creating ‘*Injury Prevention for Children and Teens*’, a course of 59 video learning segments within eight modules taught by a multidisciplinary panel of 25 nationally-recognized experts. Completion tracking and course evaluations were collected.

**Results:**

In 2.5 years, 4,822 learners from 148 countries have enrolled. Two-thirds of learners were female. Median age of learners was 31 years. For engagement, 19.3% (n = 932) of learners attempted quizzes, and 5.2% (n = 252) participated in online forum discussions. Medical professionals (n = 162) claimed an average of 13 credit hours per learner. Over 200 senior medical students have taken the SPOC.

**Conclusion:**

‘*Injury Prevention for Children and Teens*’ is a novel approach to injury prevention education that is broad, science-based, accessible, and not cost-prohibitive for a diverse group of global learners.

## Introduction

The Centers for Disease Control and Prevention (CDC) has called pediatric injuries ‘among the most under-recognized public health problems facing the USA.’ [1,2] Most medical training programs do not consistently offer pediatric injury prevention didactics to trainees [3–8]. The CDC National Action Plan highlighted the need to bring pediatric injury prevention training to a broader audience, including all those responsible for the health of children and adolescents [2]. In light of evolving technologies, changing curricula, and time restrictions, online courses have increasingly played an essential role in delivering public health education [9–12]. A Massive Open Online Course (MOOC) is a well-recognized online course format allowing large groups of learners to access educational content asynchronously at reduced or no cost. In 2018, over 100 million learners had enrolled in over 11,000 MOOCs available in a broad array of subjects from over 900 universities, and among those courses, approximately 1,000 were in ‘health and medicine’ [13]. However, none of the leading online course platforms (i.e., *ed-X, Coursera*) offered any courses on pediatric injury prevention.

The advantages of using MOOCs in the medical school curriculum have been well described, where MOOCs can be converted to Small Private Online Courses (SPOCs) designed for smaller, closed cohorts of designated learners (i.e., medical students) that are paired with additional, more in-depth instructor-facilitated discussions and readings [8,14]. What is less clear is how to create a ‘one-size-fits-all’ course for a broad range of learners course for a subject with widely differing topics. A traditional, week-by-week linear course design may be acceptable for the general learner if their goal is to learn about all the course topics. However, this design may be a barrier if the learner is only interested in specific topics that relate to their field of work or area of interest (e.g., a high-school coach interested in the subject of concussions or a child care provider interested in infant passenger safety). For the general learner, MOOC quality and completion rates are often determined by whether they perceive that they have learned what they wanted to learn, i.e., their learning experience is aligned with their personal learning goals [14]. Thus, a traditionally-designed MOOC, where the learner is obligated to complete the entire course in a ‘week-by-week’ manner, may not be optimal. There is a need for a new design approach that allows flexibility within the MOOC, allowing the learner to tailor their learning experience to align with their learning goals for a subject as broad as pediatric injury prevention.

This manuscript describes our response to a clear gap in pediatric injury prevention education by creating an online course that is broad, science-based, accessible, and not cost-prohibitive for a diverse group of global learners. We focus on the development and outcomes for ‘*Injury Prevention for Children and Teens*’ that functions as a flexible MOOC for the general learner and as a SPOC for the medical student. Using this novel approach, we demonstrate how we created a course that appeals to a broad set of learners and enables them to improve their clinical and public health practices, support families and communities, and learn strategies in injury prevention science.

## Materials and methods

### Curriculum development

Curriculum development was led by the CDC-funded University of Michigan (U-M) Injury Prevention Center (IPC) and was supported by the U-M Center for Academic Innovation (CAI), which partners with faculty to develop online learning materials. The MOOC, titled ‘*Injury Prevention for Children and Teens*,’ modeled an interprofessional collaboration between nationally-recognized UM-IPC experts and featured contributors holding primary appointments in the U-M Transportation Research Institute, Trauma Burn Center, Emergency Medicine, Psychiatry, Family Medicine, Office of Research, Sociology and Organizational Studies, Health Behavior and Health Education, School of Social Work, School of Nursing, School of Public Health, School of Kinesiology, Michigan Division of Pain Research, and C.S. Mott Children’s Hospital.

### Course design

Patients or the public were not involved in our research’s design, conduct, reporting, or dissemination plans. With support from the CAI team, IPC faculty and staff developed the curriculum, including learning objectives, outcomes, and evaluation questions. The CAI team filmed and edited video content and supported lecture transcripts with captioned audio for the Deaf/Hard of Hearing.

**Figure 1.**
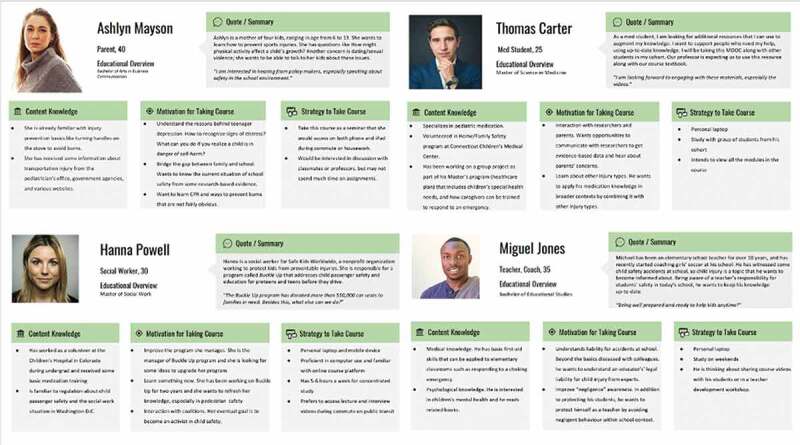
Learner personas

**Figure 2.**
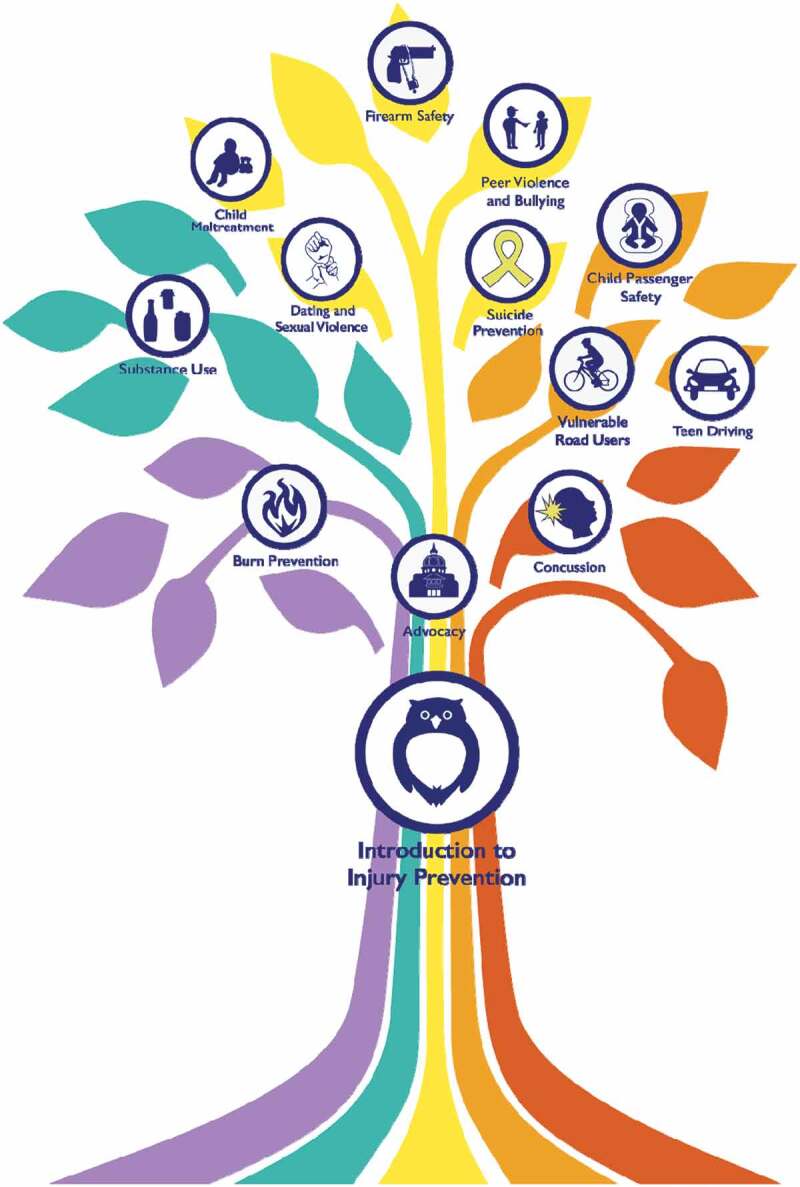
Non-linear learning dynamic

**Figure 3.**
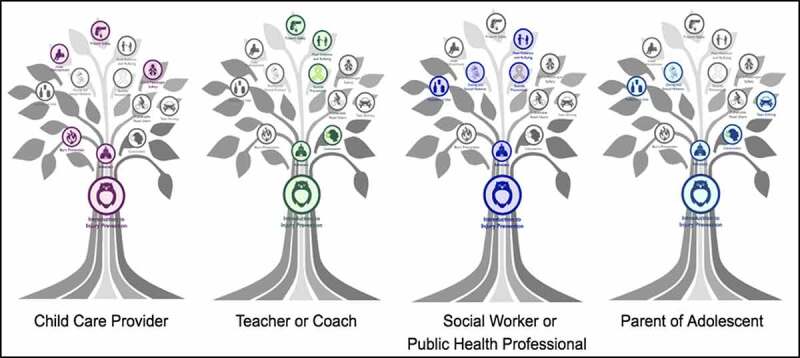
Learning pathways within the MOOC

Learner personas were developed to ensure course development was done through the lens of different learner groups (Figure 1). The MOOC was designed for a broad professional audience (medical professionals, nurses, social workers, teachers, coaches, public health students, public health practitioners). It was to be available free of charge through edX, a global online course hosting platform, and Michigan Online, an institution-specific platform [15,16]. The course was constructed so that the learner could advance through course topics nonlinearly, with pathways that could be completed in any order or combination, as illustrated in the course image of a tree with different color pathways or roots (Figure 2). All MOOC learners were required to take the ‘Introduction to Injury Prevention’ module, which forms the ‘trunk’ of the course. Afterward, however, learners could choose module topics based on individual interest using this tree figure. The course team developed ‘branch pathways,’ where specific lessons were highlighted based on developed learner profiles (e.g., teachers, coaches, parents, social workers, or medical students) (Figure 3). Essentially all general public MOOC learners could choose individual topics or branch pathways of their own liking at any time.

Pathways (from left to right): ‘Child Care Provider’, highlighting modules in Burn Prevention, Child Maltreatment, Child Passenger Safety, and Firearm Safety; ‘Teacher or Coach’, highlighting Concussion, Peer Violence and Bullying, Suicide Prevention, and Firearm Safety; ‘Social Worker or Public Health Professional’, highlighting Dating and Sexual Violence, Suicide Prevention, Peer Violence and Bullying, Substance Use; ‘Parent of Adolescent’, highlighting Concussion, Dating and Sexual Violence, Substance Use, and Teen Driving. Suggested videos and topics are depicted through a specific color scheme, while non-suggested videos or topics are greyed out.

Using the same content within the MOOC, a SPOC was then designed and created as a two-week elective for third- and fourth-year U-M medical students and a two-week elective for second- or third-year pediatric residents. Unlike the MOOC design, students within the SPOC were required to complete all modules and additional readings to earn school credit for the course. The SPOC format, different from the global MOOC, allows the instructors to teach to a particular cohort of students, adjust the difficulty or depth of the course more readily with more advanced readings, and interact with individual students within the course, while closely tracking quiz scores as students progress through the course. For example, additional assigned readings in a SPOC could differ between a SPOC designed for pediatric residents (e.g., an article on how to incorporate injury prevention science into well-child checks) vs. public health students (e.g., an article on how to conduct an injury prevention education intervention in the community).

The U-M Office of Continuing Medical Education (CME) and Lifelong Learning oversaw the CME credit approval process. The MOOC was disseminated through U-M CAI, social media platforms, numerous academic listservs, the U-M IPC listserv and website, print ads and presentations at academic conferences, and various partner and academic organizations. The SPOC was advertised to U-M medical students through internal student email listservs and social media platforms. The MOOC was also promoted to public health students as part of the U-M School of Public Health injury prevention certification program and independent study program.

### Course assessment and learner data

There were eight specific modules within the full course, with 18 machine-graded quizzes designed for periodic learner assessment with 123 multiple-choice questions. Each question allowed up to three attempts. Within the MOOC, learners only completed quizzes if they completed the module in the sequence it was designed for – each quiz within a module had pre-requisite elements. A score of 70% on an assessment was required to receive credit for each quiz and a comprehensive score of 70% to earn credit for the entire course. However, if learners wanted to earn a full course certificate, they would need to complete all assessments. A discussion board was also available and monitored for further learner engagement or questions. In the SPOC, medical students and medical residents were expected to complete quiz questions for all eight modules. Similar grading criteria were used to assign credit (pass/fail grading) for the elective.

Learner data from both the MOOC and the SPOC were captured by the learning platform registration data and exit surveys, including the number of learners accessing the course, the number of accessed videos, the geographical location of learners, their age, and their level of educational attainment. In addition to the learning platform data, the team developed evaluations for CME-seeking learners and non-CME seeking learners using the MOOC. MOOC module evaluations, available as survey links upon module completion, were voluntary for non-CME-seeking learners. In contrast, CME-seeking learners were required to complete online module evaluations (as a survey link) at the end of modules to receive CME certification. Both voluntary and anonymous formal assessments of the SPOC were conducted through the medical school. The University medical school Institutional Review Board (IRB) expert deemed the use of anonymous and pooled evaluation data as quality assurance and quality improvement non-clinical activity with the intent of evaluating and existing programs and therefore classified as a ‘not regulated’ IRB project.

## Results

### Final product

**Figure 4.**
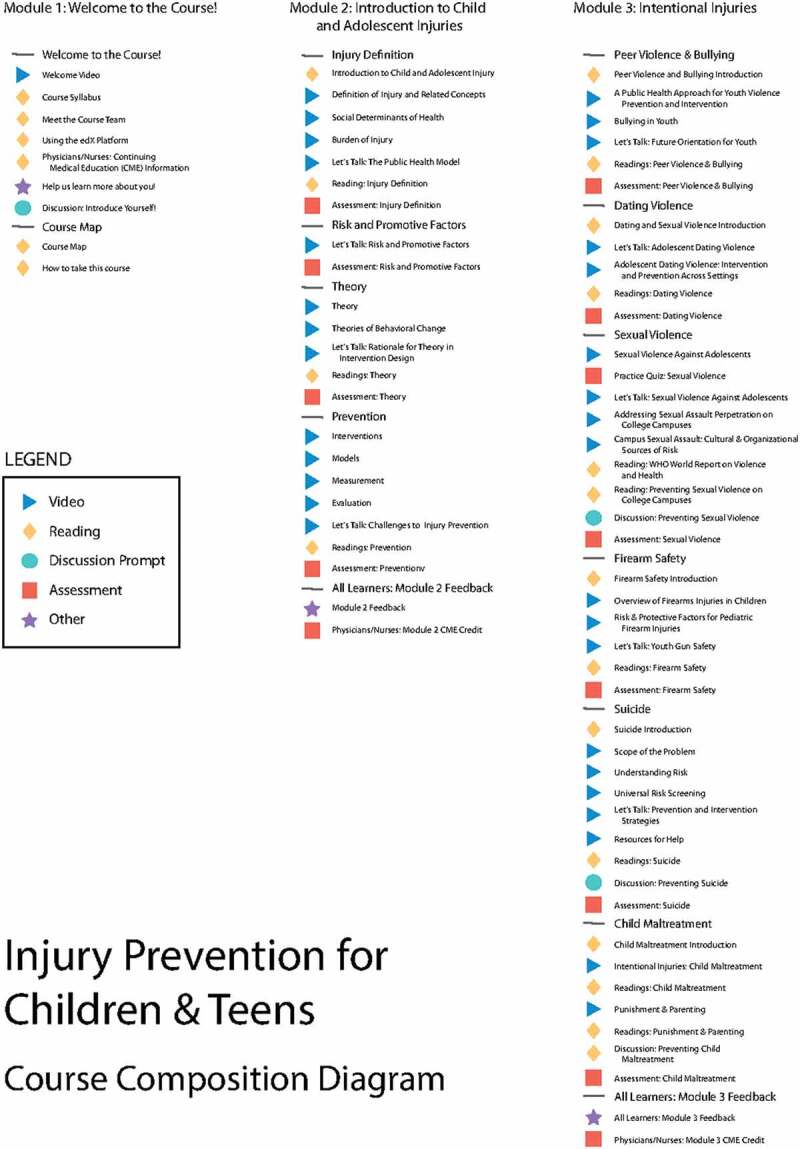
Course outline

The ‘*Injury Prevention for Children and Teens*’ course was officially launched on April 24th, 2018 on the edX learning platform. The final MOOC and SPOC courses contained 59 video learning segments divided into eight modules comprising 42 lectures, five demonstrations, and 12 expert interviews (Figure 4), with an average video length of 11 minutes. The Medical School CME Office approved the MOOC for a range of 1 to 9.75 CME credits (depending on module) that could be claimed for each module completed or a maximum of 25.5 CME total earned credits if the entire course was completed.

### MOOC learner demographics

**Figure 5.**
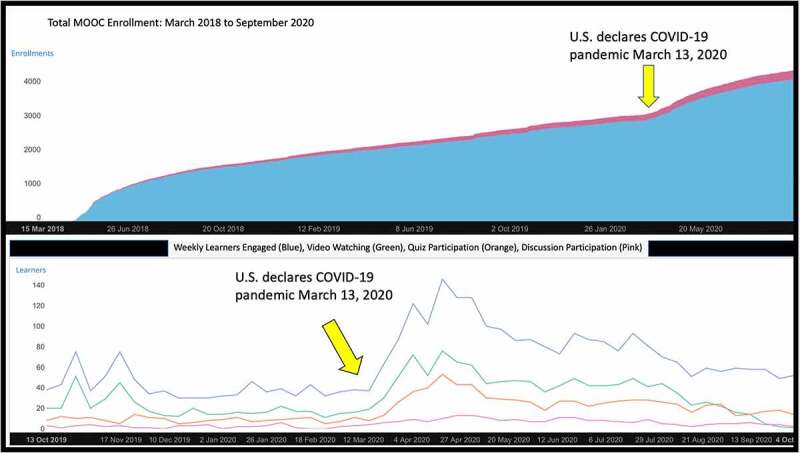
MOOC enrollment and engagement amid the COVID-19 pandemic

In the two and half years since its launch (04/24/18 – 09/30/20), 4,822 learners enrolled in the MOOC from 148 countries. Of the learners, 66.2% were female. The median age of learners was 31 years old, and the majority of learners were between 26 and 40 years of age (30.2% were 25 years old or under, 48.6% were 26 to 40 years old, and 21.2% were 41 years old or over). For achieved level of education, 24.1% of learners held a high school diploma or less, 43.6% had college degrees, and 28.7% had advanced degrees. In terms of engagement, 932 (19.3%) learners attempted quiz questions, and 252 individuals (5.2%) participated in at least one-course discussion. The most viewed module after the welcome module was Module-2: ‘Introduction to Child and Adolescent Injuries’, followed by Module-3: ‘Intentional Injuries’. Medical professionals (n = 162) claimed over 2086 credit hours for an average of 12.9 credits per medical professional. Our course demonstrated a significant uptick in enrollment and engagement at the start of the U.S. COVID-19 pandemic (Figure 5). The course averaged ~80 new learners a week during this time, compared to about 20 new learners per week in the weeks before COVID-19.

### MOOC learner feedback

A total of 824 evaluation surveys were completed by learners that did not request CME, and 512 surveys were completed by 162 unique learners seeking CME. The majority (91.2%) of survey responses found the presentations to be relevant to their work (CME = 94.5%, Non- CME = 89.1%), and 72.3% felt that the course did ‘very well’ or ‘extremely well’ at addressing how to implement evidence-based injury prevention best practices (CME = 76.8%, Non- CME = 69.5%). 81.5% of survey responses evaluated the course as ‘above average’ (CME = 85.0%, Non-CME = 79.3%), and 66.3% said they were either ‘somewhat’ or ‘extremely likely’ to change their practice (CME = 69.5%, Non-CME = 64.3%). From evaluation survey responses, there were learners from many specialties and professions that completed portions of the course, including physicians (9.3%), medical residents/students (6.2%), nurses (15.4%), public health researchers/practitioners (10.7%), coaches (7.9%), and childcare providers (13.5%). Learners were also asked to provide recommendations for improving the course and suggestions for future topics. These recommendations included: adding more content on global injury prevention, including more practical examples, and updating the course with additional topics such as drowning prevention, child abuse prevention, and more early suicide interventions. Learners also requested videos be updated with new data as the course matures.

### SPOC learner demographics and feedback

Since launching in 2018, 222 medical students enrolled in the SPOC as a two-week online course elective. Among those enrollees, 48 students (22%) filled out the optional learner feedback survey. Eighty percent (n = 38) of respondents rated the course’s quality as ‘very good’ or ‘excellent’; 84% (n = 40) felt the course somewhat or significantly changed their perception of injury prevention. Almost 46% (n = 22) of respondents said they would change their medical practice due to this course. The majority of respondents (96%, n = 46) reported that the course increased their understanding of effective pediatric injury prevention and intervention strategies and 100% (n = 48) reported increased knowledge about the importance of using evidence-based injury prevention practices.

## Discussion

‘*Injury Prevention for Children and Teens*’ is a novel response to the current gap in injury prevention education. We used national priorities, such as those detailed by the CDC, to fill this educational gap [2]. The flexible online course provides a broad platform covering numerous topics and various learner groups. The course is available to learners globally, is widely available to the interprofessional community, and offers medical professionals opportunities to earn free CME. Another novel aspect of our MOOC was its flexible ‘choose-your-topic’ design, rather than a week-by-week linear course, a format that allows for more effective dissemination of broad and diverse topics, as learners can select topics that are of most interest and relevance. The nonlinear approach removes a significant barrier for learners who usually cannot access topics of interest without progressing chronologically through the entire course or taking a full semester class. This design feature also facilitates adding supplementary topics to the course without having learners return and repeat the course starting at the beginning. An additional level of added flexibility is accomplished by the ability to start and stop lessons. The short nature of the videos facilitates completing modules between other time commitments, and the course can be taken anywhere there is an internet connection.

**Figure 6.**
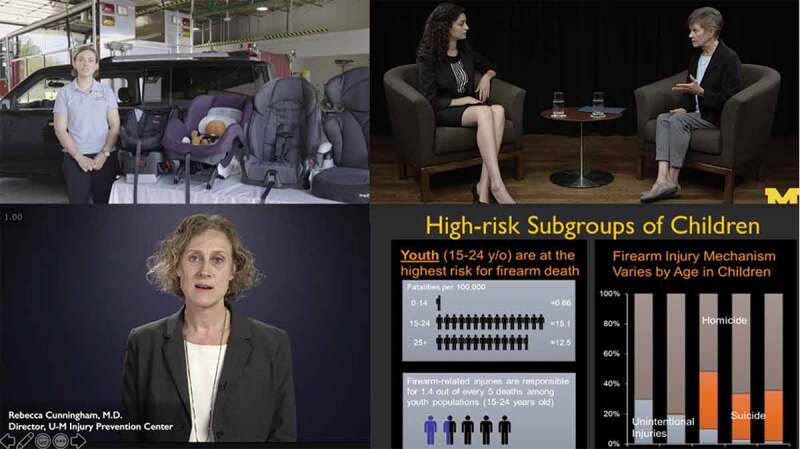
MOOC format examples

Online education presents an opportunity to use evidence-based methods and re-imagine how to best deliver lessons, enhance engagement, and optimize knowledge retention. ‘*Injury Prevention for Children and Teens*’ uses various media, including interview-format conversations with national content experts, customized slide decks with voice-overs, demonstrations, and group-table interviews (Figure 6). The varied education delivery vehicles are short in length to facilitate focus and are accompanied by frequent quizzes for meaningful engagement with the prior lessons and underscoring key takeaways. While the impacts of the COVID-19 pandemic were widespread, the transition to national shelter-in-place orders quickly underscored the value of effective distance learning and online education for students across grades and disciplines. Nationwide, schools and universities transitioned to online learning. New online courses were developed quickly on short timelines to continue providing education to learners, even within the medical field [17]. High-quality pre-existing online courses, such as MOOCs and SPOCs, are valuable educational resources when flexibility is essential, as demonstrated during the pandemic.

Four stills from the online course demonstrating the several ways that education is delivered, through demonstrations (upper left), interviews (upper right), lectures (bottom left), and didactics with voiceovers (bottom right). Permissions have been obtained from all people depicted in the images, who are either manuscript co-authors or course instructors.

CME leads to an improvement in clinician performance and, to a lesser extent, improved patient outcomes [18]. However, traditional educational venues, such as conferences, present a financial barrier of entry. Resource-poor areas, both domestically and internationally, have fewer opportunities for CME compared to resource-rich settings. However, our course has the opportunity to reach rural areas, where a disproportionate amount of injury occurs. Increased access to online injury prevention education addresses the substantial travel barrier for rural learners. Online courses in medical education are increasingly being developed and accepted for CME credit [9,19–21]. Existing MOOCs are often free to enroll, but may require a small fee to validate completion, provide a certificate, or qualify for CME [9]. Our goal was to create an entirely free MOOC that was accessible and widely-used. Reducing the financial barrier to CME credits encourages medical professional involvement and serves to widely disseminate pediatric injury education.

In addition to accessible CME opportunities, there is also a need for pediatric injury prevention curricula for the global international audience [5]. Other MOOCs have been created previously for international purposes, such as during the Ebola crisis [22,23]. MOOCs, especially when widely distributed, become increasingly cost-effective [12]. Our course has been taken by a large cohort of international learners, providing novel access to injury prevention education in high-need areas.

Our course has limitations. Although it covered a wide range of pediatric injury prevention topics, it did not cover drowning and choking topics. Also, unlike other courses that may be narrower in scope (e.g., Ebola), our course will require more effort to ensure its data and content remain relevant. While our course offered CME for physicians and nurses, we did not yet provide credit for other healthcare professions (e.g., social workers). Course feedback was voluntary, and thus survey responses from students and learners were limited. Additionally, our course did not have a long-term follow-up mechanism to assess learners’ perceived practice changes. Although our MOOC was accessed internationally, the course content was not designed specifically for the international audience and may not address some of the injury topics that might be more prevalent or different in developing low-middle income countries.

## Conclusion

The ‘*Injury Prevention for Children and Teens*’ online course is unique in its delivery while filling a critical gap in offering injury prevention education regionally, nationally, and globally. Dual-structure (MOOC/SPOC) online courses such as this are a model for addressing systemic deficiencies in medical and public health education. Our MOOC offers additional flexibility allowing learners to design their own educational experience. Our focus on diverse media formats, short lecture segments, frequent assessments, and discussion boards reflects a shift in how education is best delivered to accommodate different learners’ needs. Using CDC data, content creators can identify areas of highest need and continue this new wave of tailored and flexible injury prevention education, where MOOCs can lead the way.
